# Longitudinal Prevalence of Antibodies to Endemic Pathogens in Bulk Tank Milk Samples From Dairy Herds Engaged or Not in Contract Heifer Rearing

**DOI:** 10.3389/fvets.2021.785128

**Published:** 2021-11-25

**Authors:** Marie-Claire McCarthy, Luke O'Grady, Connor G. McAloon, John F. Mee

**Affiliations:** ^1^Teagasc, Animal and Bioscience Research Department, Dairy Production Research Centre, Fermoy, Ireland; ^2^School of Veterinary Medicine, University College Dublin, Dublin, Ireland

**Keywords:** contract heifer rearing, ELISA, bulk tank milk sampling, dairy herds, endemic infectious disease

## Abstract

Since the abolition of EU milk production quotas in 2015, Europe's dairy industries have undergone a period of rapid expansion with possible resultant increased inter-herd transmission of endemic pathogens. The aims of this study were (1) to establish the post-2015 prevalence of antibodies to selected endemic infectious diseases and (2) to determine if prevalences differed between herds where heifers were reared at home and those where heifers were sent out for contract-rearing. Three bulk tank milk (BTM) samples were collected annually between May and August of 2018–20 inclusively from 120 Irish dairy herds. Additionally, herd vaccination status was collected by questionnaire. Milk samples were tested using commercially available ELISAs for eight pathogens: bovine viral diarrhea virus (BVDV), bovine herpesvirus 1 (BoHv-1), bovine respiratory syncytial virus (BRSV), *Mycoplasma bovis, Mycobacterium* a*vium* subspecies *paratuberculosis* (MAP), *Salmonella* Dublin (*S*. Dublin), *Leptospira* Hardjo (*L*. Hardjo), and *Neospora caninum (N. caninum)*. The true prevalence of each pathogen was calculated using a Rogan-Gladen estimator. The true prevalences (95% CI) of BTM antibodies in unvaccinated herds across the 3 years were as follows (i) BVDV: 57, 86, and 73% (95% CI: 40.7–65.9, 74–94, and 58–85) (*n* = 56, 56, and 48), (ii) BoHv-1: 47, 49, and 19% (95% CI: 26.3–69.7, 25–75, and 1–56) (*n* = 21, 20, and 11), (iii) *L*. Hardjo: 34, 59, and 73% (95% CI: 12.5–63, 33–82, and 33–99) (*n* = 15, 21, and 10), (iv) *S*. Dublin 32, 57, and 11% (95% CI: 12.21–68.1, 30.2–90.1, and 0) (*n* = 19, 22, and 13), (v) BRSV: 100% (95% CI: 99.5–100, 100, and 100) (*n* = 120, 109, and 91), (vi) MAP: 0% (95% CI: 0, 0, and 0) (*n* = 120, 109, and 91) (vii) *N. caninum* 0% (95% CI: 0, 0, and 0) (*n* = 120, 109, and 91) and (viii) *M. bovis* (ELISA) 53, 0.42, and 30% (95% CI: 3.95–6.84, 0, and 21–41) (*n* = 120, 109, and 91). *M. bovis* was detected by PCR in 0, 1, and 0% of herds in 2018, 2019, and 2020, respectively. This study showed that expanding Irish dairy herds are endemically infected with several of the studied pathogens. No differences in herd prevalence of infectious agents were observed between farms with different heifer rearing strategies (contract-rearing vs. traditional rearing).

## Introduction

Several infectious diseases are endemic in the Irish cattle population including bovine viral diarrhea (BVD), Johne's disease (MAP), infectious bovine rhinotracheitis (IBR), bovine respiratory syncytial virus (BRSV), tuberculosis, neosporosis, mycoplasmosis, salmonellosis and leptospirosis (1, 2). The economic implications of these infections for dairy producers are significant due to reduced milk production, infertility, increased mortality, treatment costs, premature culling, and increased replacement rate (3). Furthermore, antibiotic usage associated with management of these infectious diseases may contribute to the growing problem of antimicrobial resistance (4).

Previous Irish studies have estimated the prevalence of herd exposure to BVDv, BoHv-1, *Salmonella* spp., *Neospora caninum* (*N. caninum*), and Leptospira *hardjo* (L. *Hardjo*) at 80, 78, 49, 19, and 86%, respectively, in unvaccinated dairy herds using BTM analysis (5, 6). Using serological testing, the ELISA true prevalence of Johne's disease among Irish dairy herds has previously been estimated 20.6% (7). All of these studies were carried out prior to the abolition of EU milk quota in 2015. Milk production quotas were initially imposed on EU member states in 1984 to address the oversupply of dairy products to the EU market and the associated volatility in milk price associated with this overproduction. As a result, the aim of the quota system was to limit expansion of dairy production systems within member states. Increased international demand for dairy products, particularly in developing economies, resulted in complete removal of milk production quota in 2015, facilitating unhindered expansion of dairy herds. Since 2015, Irish dairy farmers have responded to expansion opportunities by increasing the size and specialization of their farms (8). As Irish dairy herds continue to grow, often by purchase of animals, maintaining herd biosecurity represents a considerable challenge for herd owners. As a precedent, a US survey of dairy producers (9) found that all respondents engaged in herd expansion experienced increased losses associated with one or more of the following infectious diseases; BVD, Johne's disease, and IBR as a result of breakdown in herd biosecurity practices (9).

In addition, national prevalence data for *Mycoplasma bovis* (*M. bovis*) among Irish dairy farms are not available. Thus, the prevalence of this suite of infectious pathogens has not previously been reported and the data that are available precede dairy herd expansion.

Contract-rearing of replacement heifers is proposed as a potential solution to overcome the land and labor resource shortages associated with the recent rapid expansion of Ireland's dairy industry. By its nature, contract-rearing involves the movement of animals between herds, with resultant increased opportunities for direct and indirect animal contacts and potential for infectious disease transmission between heifers returning from the rearing unit and cows on the source dairy farm.

The aim of this study was therefore to determine the most recent post-quota, herd-level prevalence of eight endemic pathogens (BVDv, BRSV, MAP, BoHv-1, *N. caninum, M. bovis, S*. Dublin, and *L*. Hardjo*)* in Irish dairy herds using bulk tank milk samples. A further objective of this study was to compare the prevalence of these pathogens among dairy herds where replacement heifers are reared off-site (contract-reared) to those where heifers are reared on-site.

## Materials and Methods

### Study Population

**Figure 1:**
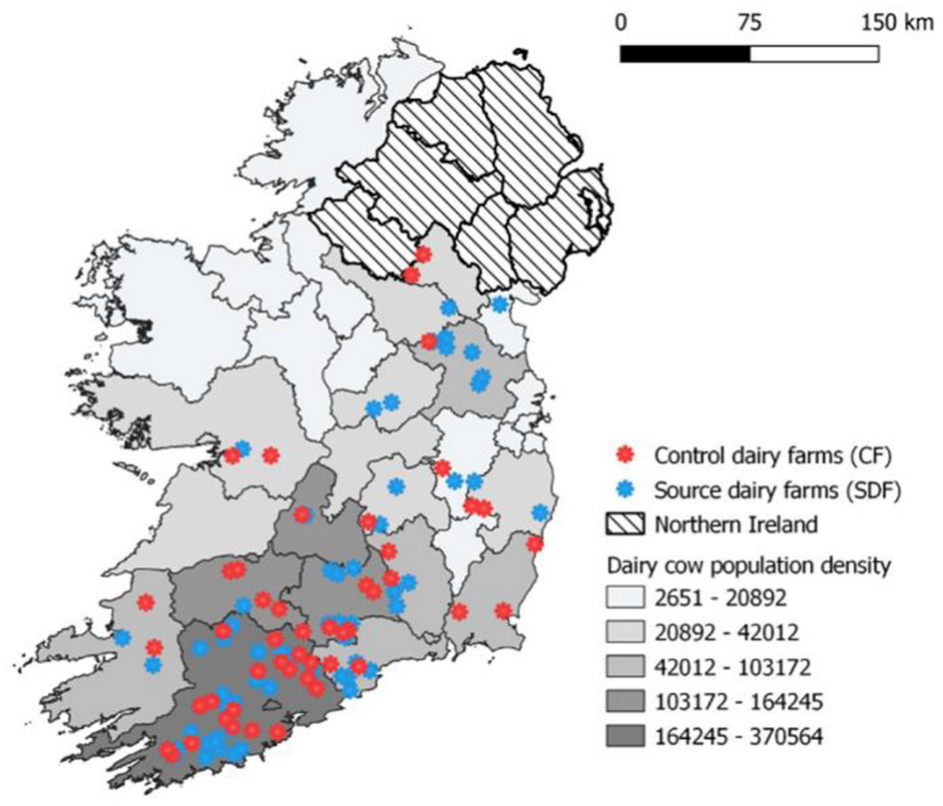
Location of study herds and density of dairy cow population in the Republic of Ireland during 2018.

Herds were recruited to this study as described by McCarthy et al. (10). Briefly, through a multistep process involving the national cattle breeding database and several Irish farming stakeholder bodies and media awareness campaigns, 120 dairy farmers were recruited into a wider 3-year nationwide longitudinal study to investigate the risks to animal health associated with contract heifer rearing. Of these farmers, 65 were sending heifers off-site for rearing purposes (SDFs, source dairy farms) and the remaining 55 were rearing heifers on their farm of origin (CFs, control farms). Surveyed farms were distributed across all four provinces of Ireland, with the largest density of farms located in Munster, reflecting the national dairy cow population distribution (Figure 1). In 2018 in Ireland, there were ~18,000 dairy farmers (11), and the recruited farms represent <1% of the national dairy farmer population. The majority of study herds were classified as spring-calving (92%), with remaining herds operating a split-calving pattern (spring and autumn). The predominant breed types within these herd were Holstein, Friesian, and Jersey crossbreds. The median size of study herds in 2018 was 141 cows (range 60–501), larger than the average Irish dairy herd of 86 cows at the time of herd recruitment in 2018. The majority of farmers (92% of CFs and 93% of SDFs) had increased their herd size between 2013 and 2018. Within the two farmer cohorts, source dairy herds (median herd size 195 cows, range 60–380) were larger than control herds (median herd size 120.5 cows, range 62–501) (10).

### Sample Collection and Analysis

Between 2018 and 2020, participating farmers submitted three bulk tank milk samples by post using a standardized sampling kit. Sampling kits were posted to farmers annually during May, corresponding to the period immediately following the end of the spring calving period when the majority of cows were lactating and thus represented in the bulk tank sample in these seasonally calving dairy herds.

Each kit contained a cover letter, sampling instructions, submission form, vaccination protocol questionnaire, a 250 ml jug and two 50 ml sample tubes, each containing a preservative tablet (Broad spectrum Microtabs 2, D & F Control Systems Inc., USA). Farmers were requested to return samples on the day of sample collection by post within 2 weeks of receiving the kit, after which weekly reminder text messages were sent to non-responders. Follow up phone calls were made to farmers who did not return samples after one month. Samples were placed in storage at −20°C immediately upon arrival at the research center. At the end of the sampling period, samples were collectively transported to a commercially accredited laboratory for testing (FarmLab Diagnostics, Roscommon, Ireland).

Commercially available ELISA kits were used to test milk samples for the presence of antibodies to BVD, *Leptospira* Hardjo, *Salmonella* Dublin, *Neospora caninum*, BoHv-1, *Mycobacterium avium* subsp. *Paratuberculosis* (MAP), *Mycoplasma bovis* and BRSV (Table 1). Additionally, the presence of *M. bovis* antigen was established using a real-time polymerase chain reaction (RT-PCR) protocol on all BTM samples [cycle threshold (Ct) cut-off value of 37 (12)]. All tests were conducted according to the manufacturers' instructions. Where an ELISA kit with superior sensitivity (Se) or specificity (Sp) became available during the study period, these kits were used in preference to kits with lower Se/Sp used on samples from the early study period (BVDV and *M. bovis* ELISA kits).

**Table 1:** Details of ELISA kits used to test bulk tank milk samples from study herds between 2018 and 2020.

**Pathogen**	**Year used**	**ELISA test kit name**	**ELISA test kit manufacturer**	**Reported sensitivity (%)**	**Reported specificity (%)**	**Pp cut-off value used**
BVDV	2018	BVDV p80 Ab	IDEXX Laboratories, Westbrook, MA	93.6	100	0.2
	2019, 2020	ID Screen® BVD p80 Antibody Competition	IDVet, Montpellier, France	100	100	>65
*L*. Hardjo	All sampling periods	Linnodee Leptospira Hardjo ELISA	Linnodee Ltd., Ballyclare, Northern Ireland	94.1	94.8	>0.1
*S*. Dublin	All sampling periods	ID Screen® *Salmonlla* Dublin milk	IDVet, Montpellier, France	63.2^*^	99.1	≥70
*N. caninum*	All sampling periods	ID Screen® Neospora caninum milk	(IDVet, Montpellier, France)	88	89.4	≥30
BoHv-1	All sampling periods	IDEXX IBR Pool Ab Test	IDEXX GmbH, Germany	90.7	99.2	>25
MAP	All sampling periods	IDEXX MAP Ab Test	IDEXX GmbH, Germany	56	96	>30
*M. bovis*	2018	BIO K 302 indirect Ab test	BioX Diagnostics, Belgium	60.4	97.3	≥37
	2019, 2020	ID Screen® *Mycoplasma bovis* antibody ELISA	IDVet, Montpellier, France	93.5	98.6	≥30
BRSV	All sampling periods	SVANOVIR® BRSV-Ab	SVANOVIR® BRSV-Ab ELISA, Boehringer Ingelheim Svanova, Sweden	94.6	100	≥10

* *Sensitivity information was not available for this ELISA kit. For true prevalence calculations, an Se of 63.2% from a comparable kit was used [Salmonella B/D LPS antibody ELISA (bulk milk), GD Animal Health Services, Netherlands]*.

Details relating to herd vaccination protocol were collected in a questionnaire enclosed in the milk sampling kit. Participating farmers were asked to record whether or not they were vaccinating for each infectious disease (yes/no). For farmers implementing a vaccination protocol for any given disease, further information was gathered on vaccination dates, number of doses administered, and product(s) used to vaccinate cows and heifers during each of the 3 sampling years (2018–2020).

### Data Analysis

Vaccination questionnaire data and results of laboratory testing were entered into a database worksheet programme (Microsoft Excel, 2010). Statistical analyses were conducted using SPSS24.0 (SPSS Inc., Chicago, IL). Chi-squared tests were used to test associations between herd type and true-prevalence estimates (Fischer's exact test was used when more than 20% of cells had expected frequencies of <5). A significance level of 0.05 was used.

Bulk tank milk results were dichotomised into positive or negative outcomes depending on the positive cut-off values described in Table 1. Inconclusive results were combined with negative results. The apparent prevalence of infectious agents in unvaccinated herds (where applicable) at each time point was calculated by dividing the total number of positive herds by the total number of all unvaccinated herds sampled. The true prevalence for each herd was calculated using a Rogan-Gladen estimator (EpiTools epidemiological calculator, AusVet) using the reported specificity and sensitivity of ELISA test kits outlined in Table 1 below (13–15). Eight samples from the 2020 sampling period were unsuitable for testing and these samples were excluded from the analyses conducted on samples collected during 2020.

## Results

### Study Population

Of the 120 recruited dairy farmers, 100, 92.5, and 83% returned a milk sample for testing in 2018, 2019, and 2020, respectively. Of the milk samples returned, 120, 109, and 91 samples were suitable for analysis for each period, respectively.

Rates of vaccination uptake for BVD, IBR, leptospirosis, and salmonellosis among study herds are outlined in Table 2. There was no vaccine available in Ireland for *N. caninum*, MAP, and *M. bovis*. Products available to vaccinate against BRSV in Ireland were marketed for the control of respiratory disease complex in calves, typically as multivalent vaccines. As a result, the vaccine use survey used in this study did not attempt to elicit information on BRSV vaccine use in lactating cows.

**Table 2:** Vaccination use (% of herds) among study herds for BVD, IBR, leptospirosis, and salmonellosis.

**Year**	N^*^	**BVD**	**IBR**	**Leptospirosis**	**Salmonellosis**
2018	120	53.3	82.5	88.3	84.2
2019	109	48.6	82.6	80.7	80
2020	91	47	88	89	86

* *N, number of vaccination questionnaire responses received; BVD, Bovine viral diarrhea; IBR, infectious bovine rhinotracheitis*.

Vaccine usage was generally higher on SDFs than CFs, depending on the year. Vaccination against IBR was consistently more commonly adopted by SDFs than CFs during the 3 sampling years (*p* = 0.01, 0.01, and 0.009 for 2018, 2019, and 2020, respectively). Use of a vaccination protocol for leptospirosis was greater (*p* = 0.03) among SDFs than CFs in 2019. There were no differences observed for remaining vaccination protocols across farm type.

### Prevalence

**Figure 2:**
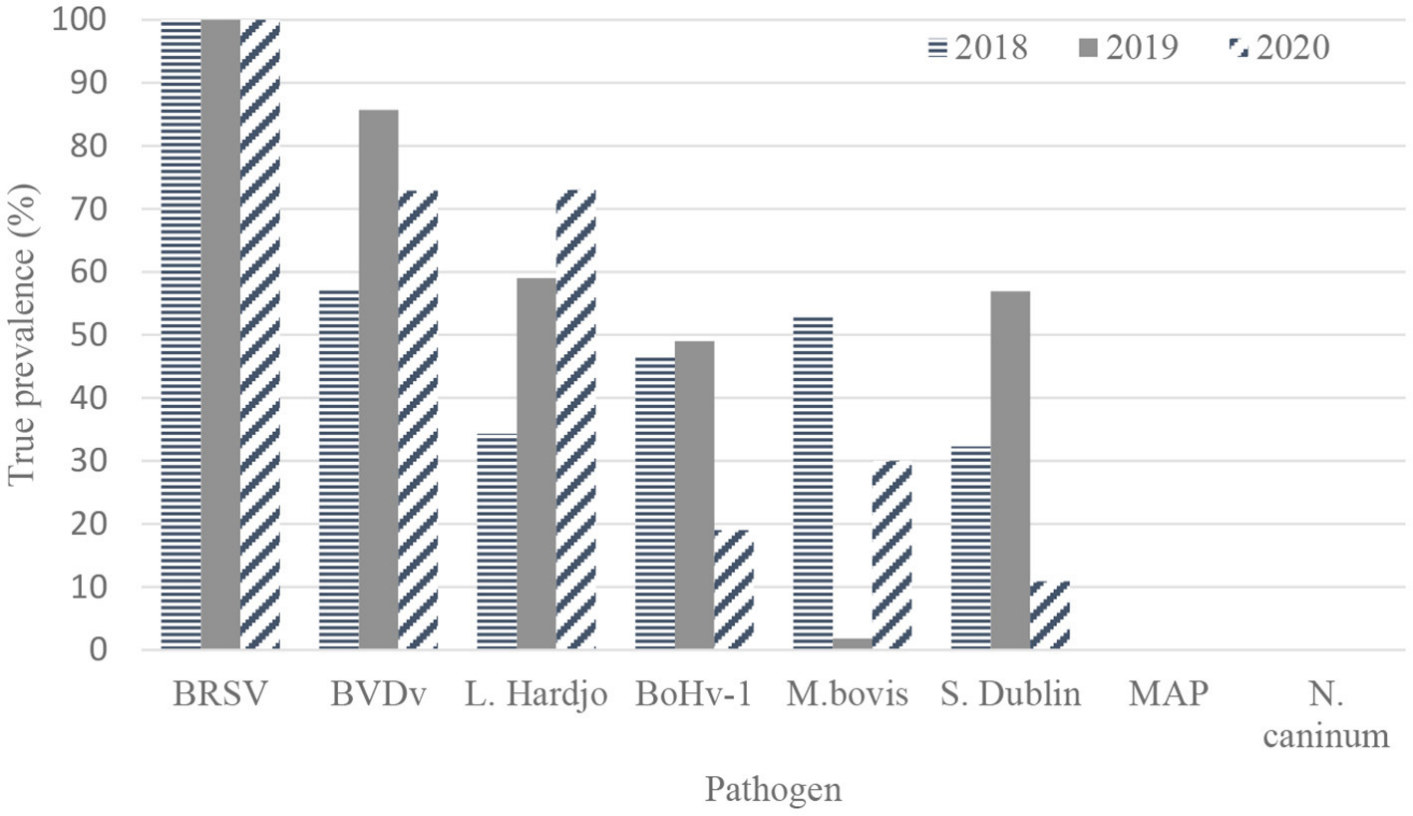
True herd-level antibody prevalence estimates for BRSV (bovine respiratory syncytial virus) (*n* = 120, 109, and 91 herds), BVDV (bovine viral diarrhea virus) (*n* = 56, 56, and 48), *L*. Hardjo (*Leptospira* Hardjo) (*n* = 15, 21, and 10), BoHv-1 (bovine herpes virus-1, *M. bovis* (*Mycoplasma bovis*) (*n* = 120, 109, and 91), *S*. Dublin (*Salmonella* Dublin) (*n* = 19, 22, and 13), MAP (*Mycobacterium avium* subspecies paratuberculosis) (*n* = 120, 109, and 91) and *N. caninum* (*Neospora caninum*) (*n* = 120, 109, and 91) based on results from bulk tank milk sampling on 120, 109, and 91 unvaccinated dairy farms in 2018, 2019, and 2020, respectively.

The true herd-level prevalences of antibodies to pathogens in unvaccinated herds are presented in Table 3 and Figure 2.

**Table 3:** True herd-level antibody prevalence estimates and 95% confidence intervals for BVDV (bovine viral diarrhea virus), BoHv-1 (bovine herpesvirus-1), *L*. Hardjo (*Leptospira* Hardjo), *S*. Dublin (*Salmonella* Dublin) BRSV (bovine respiratory syncytial virus), MAP (*Mycobacterium avium* subspecies *paratuberculosis), N.caninum* (*Neospora caninum*), and *M.bovis* (*Mycoplasma bovis*) based on results from bulk tank milk sampling on 120, 109, and 91 unvaccinated dairy farms in 2018, 2019, and 2020, respectively.

		**2018**					**2019**						**2020**					
**Pathogen**	**Overall (%)**	**95% CI (%)**	**SDF (%)**	**95% CI (%)**	**CF (%)**	**95% CI (%)**	**Overall (%)**	**95% CI (%)**	**SDF (%)**	**95% CI (%)**	**CF (%)**	**95% CI (%)**	**Overall (%)**	**95% CI (%)**	**SDF (%)**	**95% CI (%)**	**CF (%)**	**95% CI (%)**
BVD	57.2	40.7–65.9	55.5	0.86–1	58.6	0.6–0.92	85.7	74–94	89.7	0.8–1	81	0.68–0.98	72.9	58–85	79.2	0.63–0.97	66.7	0.5–0.88
BoHv-1	46.8	26.3–69.7	73.2	0.32–0.99	36.19	0.16–0.63	49	25–75	73	0.32–0.99	39	0.17–0.67	19	1–56	0	0–0.72	24	0.06–0.6
*L*. Hardjo	34.3	12.5 −63	39	0.074–0.81	31.7	0.08–0.67	59	33–82	75	0.35–0.97	51	0.24–0.76	73	33–99	100	0.43–1	59	0.22–0.88
*S*. Dublin	32.3	12.21–68.1	52	0.17–1	14.6	0–0.63	56.9	30.2–90.1	46.7	0.16–0.95	65.4	0.29–1	10.9	0	0	0–0.61	21.5	0–0.8
BRSV	100	99.5–100	100	NA	100	NA	100	100	100	NA	100	NA	100	100	100	NA	100	NA
*M. bovis*	53	3.95–6.84	54	NA	52	NA	0.42	0	2	NA	0	NA	30	21–41	29	NA	31	NA

*Overall: SDF and CF. SDF, source dairy farmers; dairy farmers sending replacement heifers off-site for rearing purposes; CF, control dairy farmers; dairy farmers rearing their own replacement heifers*.

There were no significant differences observed in herd-level prevalence among unvaccinated source and control dairy farms for all pathogens across the three sampling periods.

## Discussion

The objectives of this study were to establish herd-level prevalence of exposure to selected endemic infectious diseases among Irish dairy herds post quota abolition and secondly to compare the prevalence of antibodies to these pathogens in herds among two cohorts of dairy farmers employing different heifer rearing strategies: those sending heifers off-site to a contract-rearing facility and those rearing heifers at home. This is the first study to explore both of these issues, nationally and internationally, respectively.

### Vaccine Use

The adoption of vaccine protocols was considerably higher among farmers in this study when compared to previous Irish (16, 17), UK (18), and European studies (19). Some of this difference may be due to changes in vaccine use over time in Ireland, particularly since herd expansion in 2015, and differences between countries in vaccine adoption by dairy farmers. Additionally, due to the increased risks to herd biosecurity associated with contract-rearing of heifers, source dairy farmers may have heightened awareness of the importance of disease prevention measures, such as vaccination, when compared to farmers rearing heifers at home, hence their greater use of some vaccines, details hereunder.

### Prevalence

#### BRSV

The high prevalence of BRSV antibodies among study herds (100%) across the three sampling periods was expected, given that it is a major cause of morbidity associated with respiratory disease in calves in Ireland (20, 21). There are no comparable Irish studies on the prevalence of BRSV in BMT samples. BRSV vaccination typically forms part of multivalent bovine respiratory disease vaccines marketed for administration to youngstock in Ireland. As a result, it was difficult to establish BRSV vaccination status of lactating cows in study herds. The duration of BRSV antibody persistence post vaccination is estimated at between 8 and 12 months (22, 23), thus vaccination of youngstock is unlikely to affect antibody concentrations in lactating animals (>2 years old). As such, a positive BTM result for BRSV is likely to indicate natural infection. Risk factors for BRSV infection include purchase of animals, larger herd size and housing of youngstock separately from cows until pregnancy (24), all of which applied in these herds. Our findings are consistent with those of other European studies on BRSV prevalence (25–27).

#### BVD

True prevalence estimates for BVD antibodies among study herds were lower than expected with 57, 86, and 73% of unvaccinated herds testing positive for antibodies in 2018, 2019, and 2020, respectively. Previous Irish studies have reported a herd-level BVD antibody prevalence of 92.3% (28) and 80% (2) during 2009 using bulk tank milk analysis. These studies were conducted prior to the establishment of the national BVD eradication program in 2013 and the lower prevalence of infection in our herds may reflect the success of eradication efforts, with national herd BVD PI (persistently infected) animal prevalence falling from 11.3 to 1.1% between 2013 and 2018 (29).

Approximately half of study herds reported using a BVD vaccination protocol, considerably higher than reported vaccine use in previous Irish studies (17, 28). Increased vaccine use among our study population may be attributed to increased awareness of the potential challenges to herd biosecurity associated with herd expansion (9) and heightened awareness of BVD as an economically important disease since the launch of the national BVD eradication programme.

#### Leptospira Hardjo

The true prevalence of *L*. Hardjo antibodies among unvaccinated study herds was 34, 59, and 73% during 2018–20', respectively. These findings were lower than previous Irish estimates of true seroprevalence reported by O'Doherty et al. (5) and Leonard et al. (6), who found that 79 and 86% of herds were ELISA-positive for leptospiral antibodies in bulk tank milk samples in 2000 and 2009, respectively. However, as a large percentage of herds were implementing a *L*. Hardjo vaccination protocol (>80%), few unvaccinated herds were available for inclusion in prevalence estimations (<21 herds), reducing the power of the present study. This may also account for the high inter-year variation in true prevalence estimates.

#### BoHv-1 (IBR)

The prevalence of antibodies to BoHv-1 among unvaccinated herds in this study (47, 49, and 19% in 2018, 19, and 20, respectively) was lower than expected. Herd-level prevalence estimates of 80 and 75%, were previously reported by Sayers et al. (2) and Cowley et al. (30) using bulk tank milk sampling. As with *L*. Hardjo, most herds (>82%) in the present study were using an IBR vaccination protocol, resulting in few herds being included in prevalence calculations. The greater uptake of IBR vaccination among source dairy farmers may suggest greater awareness of biosecurity risks associated with contract-rearing practices among this cohort, such as open herd status and commingling of animals from multiple sources, known risk factors for IBR positivity (31, 32).

#### *S*. Dublin

The prevalence of antibodies to *S*. Dublin in unvaccinated herds across the three sampling periods was 32, 57, and 11% in 2018, 2019, and 2020, respectively. Estimates for 2018 and 2020 were considerably lower than those reported in previous Irish (49%) and UK studies (48%) using bulk milk tank milk analysis (5, 33). However, as with BVD, IBR and *L*. Hardjo, few salmonella-unvaccinated herds were available for analysis, particularly in 2020 (13 herds), resulting in considerable inter-year variation.

#### N. caninum

The prevalence of *N. caninum* in study herds was lower (0%) than that documented in previous Irish studies by O'Doherty et al. (5) and Sekiya et al. (34) (9 and 19%, respectively). As there is no vaccine against *N. caninum* available in Ireland, all current study herds were tested, with resultant data being broadly comparable. A within-herd *N. caninum* seroprevalence of at least 15% is required to detect antibody-positive farms using pooled milk samples (35), however, which may have resulted in underestimation of the prevalence of this pathogen at low levels in study herds.

#### MAP

The prevalence of MAP, 1.7, 0, and 0% (in 2018, 2019, and 2020, respectively) in study herds was lower than estimates from previous Irish studies using ELISA testing (20.6%) (7) and Bayesian estimates of herd prevalence (23–34%) (36). The low sensitivity of MAP ELISAs (e.g., 56% in the present study) renders test results unreliable with possible underestimation of true herd prevalence. Additionally, antibody detection in bulk milk samples is only possible in herds with a high prevalence of animals shedding MAP (37). Further to this, the age distribution of cows contributing to the bulk tank sample impacts on test sensitivity. In herds with a large proportion of cows in parities 1 and 2, a typical feature of expanding herds, prevalence may be underestimated due to the long seroconversion period associated with MAP infection (38, 39). The lower prevalence of MAP in 2019 and 2020 compared to 2018 may have arisen due to culling of infected animals from the affected herds.

#### M. bovis

*M. bovis* is being increasingly implicated in clinical and subclinical infections in Irish dairy herds since it was first identified here in 1994 (40). *M. bovis* infection is associated with a spectrum of clinical presentations including respiratory disease, mastitis, arthritis, and otitis. In the Republic of Ireland, during 2019, *M. bovis* was identified as the aetiological agent in 11% of cases of bovine respiratory disease (BRD) submitted for post-mortem examination across all age groups (41). This is the first study to quantify *M. bovis* prevalence in Irish dairy herds using BTM samples. Our results show a low PCR prevalence of *M.bovis* in study herds, with only one herd returning one positive result over the three sampling periods. When used alone, results from PCR analysis may underestimate *M. bovis* prevalence, due to intermittent shedding of the pathogen by infected animals and exclusion of milk from mastitic cows from bulk tank samples (42). As a result, we also used ELISA testing to detect *M. bovis* antibodies to give an indication of historical exposure status of study herds.

The ELISA true prevalence of *M. bovis* antibodies in bulk tank milk samples was 53, 0.43, and 30% in 2018, 2019, and 2020. The highest prevalence was recorded in 2018 using an ELISA (BioX BIO K 302 Mycoplasma bovis ELISA kit) with a reported sensitivity of 60.4%, suggesting possible under estimation of prevalence for this sampling period. For 2019 and 2020 samples, the ID Screen® *Mycoplasma bovis* antibody ELISA kit was used, with improved sensitivity (93.5%) compared to BioX kit. Despite the increased reported sensitivity of this test kit, prevalence estimates were lower for these sampling periods. Our results indicate a high herd-level *M. bovis* prevalence during the first sampling period, with subsequent declining prevalence in 2019 and 2020. The low prevalence in 2019 (<1%) was unexpected and repeat analysis was carried out on duplicate samples from all herds to verify these results and exclude the possibility of analytical error. No clear explanation of the reason for this inter-annual variation could be found in this or in published studies on *M. bovis* herd-level antibody prevalence. In one study, antibodies to *M. bovis* were detectable in bulk tank milk samples for up to 12 months following exposure (43). Given the 2018 herd-level prevalence, it was expected that antibodies would be detectable at a similar prevalence in 2019 samples. Temporal trends in *M. bovis* antibody persistence in bulk milk tank samples over consecutive years have not been studied however, and further research is warranted to determine the significance of these findings.

### Farm Type and Pathogen Prevalence

Given the increased biosecurity risks associated with contract-rearing of replacement heifers, it was hypothesized that the prevalence of some or all pathogens would be higher on source dairy farms engaged in contract-rearing than on farms where heifers remain on their farm of origin. By their nature, source dairy farms are open herds, with bidirectional movement of animals between the rearing and milking units increasing opportunities for exposure of heifers or milking cows to novel pathogens. Typically, heifers are moved to rearing units around the time of weaning, corresponding with a period of stress with resultant immunosuppression and increased susceptibility to infectious disease (44). Commingling opportunities at rearing units further place heifers at risk of exposure to infectious agents (45). As a result, in the absence of appropriate risk mitigation strategies, such as quarantine and testing, heifers returning from the rearing unit pose a major threat to the health of naïve cows in the milking herd. However, we found no association between farm type and prevalence of antibodies to any of the pathogens tested. Due to the small number of unvaccinated herds available for prevalence analysis, however, it is difficult to draw firm conclusions from the lack of association between farm type and herd exposure status. These findings are congruent with associated findings on biosecurity measures adopted on each farm type (10).

While this is a novel study in both updating our knowledge on prevalence of endemic pathogens and on the effects of dairy farm enterprises on same, the study had some limitations. Participation was voluntary and non-incentivised, but study herds were on average, larger than the average Irish dairy herd size of 86 cows. This resulted in a biased sample of dairy herds; however, these will be the type of herds that will predominate in Irish dairying in the future as herd size continues to grow year-on-year. The rate of vaccine use among study herds was considerably higher than reported by Sayers et al. (2) and O'Doherty et al. (5), suggesting more progressive herd owners with a greater understanding of disease prevention measures were enrolled. The level of vaccine usage by study farmers also greatly reduced the number of herds for which prevalence data could be established, resulting in lower precision of prevalence estimations.

The herd prevalence estimates reported in this study are based on testing of bulk tank milk samples for the presence of antibody or antigen. Imperfect sensitivity (Se) or specificity (Sp) associated with the test kits used may have resulted in misclassification bias and thus, over or underestimation of herd prevalence. Taking a single bulk milk sample over the course of each lactation also reduces overall sensitivity of the tests performed, particularly for pathogens present at low levels within a herd. While every effort was made to ensure samples were taken from herds within the shortest possible time frame (within 1 month of postage of sampling kit), delayed return of samples by a small number of study participants may have resulted in variability in the stage of lactation across farms at the time of sample collection. For some of the test pathogens, including *Neospora*, production of antibodies may rise as the lactation progresses (5), making comparison between herds sampled at distinct time points more difficult. Additionally, for several infectious agents, the level of antibodies is inversely related to the amount of milk produced and the dilution effect of increased milk yield is greatest ~9 weeks post-calving at peak lactation in spring-calving dairy herds such as those that predominate in Ireland (46). This corresponds to the timeframe for sample collection in this study and may result in underestimation of herd prevalence for some pathogens. This dilution effect may be further compounded by the larger than average herd size of study farms.

## Conclusions

Results from this study demonstrate that large, expanding Irish dairy herds are endemically infected with BRSV, BVDV, BoHv-1, *S*. Dublin, *M. bovis*, and *L*. Hardjo. Low herd prevalence of MAP and *N. caninum* was observed. Uptake of vaccination was higher on study farms than in previous Irish studies. No association was found between farm type (rearing heifers at home or off-farm) and exposure status for test pathogens.

## Data Availability Statement

The raw data supporting the conclusions of this article will be made available by the authors, upon reasonable request.

## Ethics Statement

The animal study was reviewed and approved by the TAEC. Written informed consent was obtained from the owners for the participation of their animals in this study.

## Author Contributions

JFM designed the study. M-CM organized the sample collection and testing, carried out the statistical analyses, and wrote the first draft of the manuscript. All authors participated in the redrafting of the manuscript.

## Funding

This research postgraduate (M-CM) was funded by the Teagasc Walsh Scholarship fund. The research work was funded as part of Project MKAB0146.

## Conflict of Interest

The authors declare that the research was conducted in the absence of any commercial or financial relationships that could be construed as a potential conflict of interest.

## Publisher's Note

All claims expressed in this article are solely those of the authors and do not necessarily represent those of their affiliated organizations, or those of the publisher, the editors and the reviewers. Any product that may be evaluated in this article, or claim that may be made by its manufacturer, is not guaranteed or endorsed by the publisher.
